# Individual with Subclinical Atherosclerosis Have Impaired Proliferation of Blood Outgrowth Endothelial Cells, Which Can Be Restored by Statin Therapy

**DOI:** 10.1371/journal.pone.0099890

**Published:** 2014-06-23

**Authors:** Javier Martin-Ramirez, Maayke G. M. Kok, Menno Hofman, Ruben Bierings, Esther E. Creemers, Joost C. M. Meijers, Jan Voorberg, Sara-Joan Pinto-Sietsma

**Affiliations:** 1 Department of Plasma Proteins, Sanquin-AMC Landsteiner Laboratory, Amsterdam, The Netherlands; 2 Department of Vascular Medicine, Academic Medical Center, Amsterdam, The Netherlands; 3 The Heart Failure Research Center, Academic Medical Center, Amsterdam, The Netherlands; 4 Department of Experimental Vascular Medicine, Academic Medical Center, Amsterdam, The Netherlands; 5 Department of Clinical Epidemiology, Biostatistics and Bioinformatics, Academic Medical Center, Amsterdam, The Netherlands; European Institute of Oncology, Italy

## Abstract

**Background:**

To study the regenerative capacity of the endothelium in patients with coronary artery disease (CAD), we cultured blood outgrowth endothelial cells (BOECs) of patients with premature CAD and their first degree relatives (FDR). Additionally we evaluated the influence of statin treatment on circulating BOEC precursors in subjects with subclinical atherosclerosis.

**Methods and Results:**

Patients with premature CAD (men <51 yr, women <56 yr) and their FDRs were included. Based on coronary calcification (CAC) scores FDRs were divided in a group of healthy subjects (CAC = 0) and subjects with subclinical atherosclerosis (CAC>0). We did not observe differences in the number of BOEC colonies and proliferation between premature CAD patients and FDRs. FDRs with subclinical atherosclerosis had lower colony numbers compared with healthy FDRs, however this was not statistically significant, and BOEC proliferation was significantly impaired (OR = 0.45, 95% CI 0.21–0.96). Unexpectedly, the number of BOEC colonies and BOEC proliferation were similar for premature CAD patients and healthy FDRs. Since a considerable number of premature CAD patients used statins, we studied the number of BOEC precursors as well as their proliferative capacity in ten individuals with subclinical atherosclerosis, before and after statin therapy. Interestingly, FDRs with subclinical atherosclerosis showed a significant increase in the number of BOEC colonies after statin therapy.

**Conclusion:**

BOEC proliferation of subjects with subclinical atherosclerosis is impaired compared with healthy controls. In these subjects, statin therapy significantly increased the number of circulating BOEC precursors as well as their proliferative capacity, revealing a beneficial effect of statins on endothelial regeneration.

## Introduction

Cardiovascular disease (CVD) represents a major health issue worldwide [1]. Endothelial dysfunction, resulting in atherosclerotic plaque formation and subsequent ischemia is the main underlying cause of this disease [2]. Circulating endothelial progenitor cells (EPCs), have been implicated in vasculogenesis in a large number of experimental studies. Their vasculogenic properties make them attractive for treatment of CVD [3]. Levels of circulating EPCs are lower in patients with coronary artery disease (CAD) than in healthy controls [4] and it has been proposed that circulating EPC levels might be a useful diagnostic tool to identify patients at increased cardiovascular risk [5], [6].

EPCs represent a heterogeneous population of circulating mononuclear cells [3], among which two distinct populations can be distinguished [7]: early endothelial progenitor cells (eEPCs) and blood outgrowth endothelial cells (BOECs), also designated as endothelial colony forming cells (ECFCs) [8], [9]. Despite their late onset, BOECs display an enhanced proliferative capacity compared with eEPCs [8]. Comparative genetic and phenotypic analyses have firmly established that eEPCs display a gene-transcription profile similar to cells of the hematopoietic lineage, whereas BOECs belong to the endothelial lineage. This implies that the regenerative capacity of the endothelium is crucially dependent on the frequency of these “true EPCs” in the circulation [7], [9], [10].

At present, limited information exists about the relation between BOECs and cardiovascular risk. We hypothesized that subjects with premature CAD or subclinical coronary atherosclerosis, as assessed by coronary CT-scanning, have lower levels of circulating BOECs and that proliferation of these cells is impaired compared with healthy controls. Besides, since it is known that statin therapy results in a rise in EPC levels [11], [12], we additionally wanted to investigate whether this also holds true for circulating BOECs. Therefore, we studied the effect of statin therapy on circulating BOECs in FDRs with subclinical atherosclerosis.

## Methods

### Subjects

Between June 2011 and June 2012 all consecutive premature CAD patients that visited the outpatient clinic at the Academic Medical Center in Amsterdam, the Netherlands, were included in this study. Premature CAD was defined as either an acute myocardial infarction or coronary atherosclerosis requiring revascularization by percutaneous coronary intervention or coronary artery bypass grafting, before the age of 51 in men and 56 years in women in line with the GENECARD definition [13]. Asymptomatic FDRs of patients with premature CAD also visited the outpatient clinic for risk assessment. All FDRs over the age of 30 years underwent coronary CT scanning to obtain a coronary calcium score (CAC), as a marker of subclinical atherosclerosis. Based on the CAC score, these subjects were divided in two groups. The first group consisted of subjects with a coronary calcium score of zero and subjects below the age of 30 in whom no CT-scanning was performed. These subjects were considered to be healthy FDRs. The second group consisted of all subjects with a positive CAC score, representing FDRs with subclinical atherosclerosis. For this study we did not perform a power calculation, since data on BOECs in CAD patients is limited. However, we assume that differences will be similar to those found in levels of early EPCs between CAD patients and healthy controls [14]. Therefore, an inclusion period of one year should be sufficient to reach significant differences between the groups.

### Ethics statement

This study complies with the Declaration of Helsinki and was approved by the ethics committee of the Academic Medical Center in Amsterdam, the Netherlands. All subjects gave written consent.

### Assessment of cardiovascular risk factors

Clinical risk factors in premature CAD patients and FDRs were assessed at the outpatient clinic. In short, all participants were questioned about their medical history, family history, symptoms of coronary artery disease and lifestyle habits. Hypertension was defined as the use of anti-hypertensive medication or in untreated individuals as a systolic blood pressure >140 mmHg and/or a diastolic blood pressure >90 mmHg. We defined hypercholesterolemia as the use of lipid-lowering medication or a fasting total cholesterol level above 6.2 mmol/L [15]. Both definitions are according to the third report of the National Cholesterol Education Program [16]. Subjects were considered smokers if they were current smokers or when they quitted smoking within the last 5 years.

In patients these characteristics were determined from data obtained by their treating physician before their cardiovascular event. Venous blood was drawn after a 12 hour overnight fast for clinical laboratory measurements.

### Coronary CT scanning

We performed a coronary CT scan in all FDRs to assess CAC, as a marker for subclinical atherosclerosis. A 64-slice multidetector CT scanner (Philips Medical Systems, Best, The Netherlands) was used to perform the CT scans. The scanning protocol was as follows: tube voltage, 120 kV; tube current, 55 mAs; detector collimation, 40×2.5 mm; gantry rotation 420 ms. Data were transferred to a post-processing workstation (Extended Brilliance Workplace; Philips Medical Systems). CAC was recorded for the main arteries; we calculated the total score by summing lesion scores of all sections.

### BOEC-colony isolation, propagation and cryopreservation

Heparinized blood samples were collected from all participants and were processed within four hours. BOEC were isolated according to a recently published protocol [17]. Multi-well 48 plates (Thermo-Scientific, Bremen, Germany) pre-coated with collagen type I were seeded with 1.5×10^6^ peripheral blood mononuclear cell (PBMCs) per well in EGM2 medium. Medium was changed three times per week. Plates were monitored at weekly intervals and wells that were filled with colonies with a cobblestone-like morphology were counted on days 7, 14, 21 and 28. As soon as a colony appeared and became 30–70% confluent, it was transferred to plates with 1.88-cm^2^ culture wells (MW-24, Thermo-Scientific) and upon confluency further propagated in 10 cm^2^ and 75 cm^2^ culture flasks. Subsequently, cells were harvested by trypsinization. 10 ml of EGM2 was added and cells were transferred to a 50 ml conical tube for centrifugation (5 min. 290*g* at 4°C). Cells were washed twice with EGM2 and slowly resuspended in fetal bovine serum supplemented with 5% DMSO (vol/vol). Cryovials were deposited in freezing containers (Thermo Scientific) which were stored at −80°C overnight. Next day cryovials were transferred to containers with liquid nitrogen. A maximum of 3 BOEC colonies per individual were cryopreserved. The ability to cryopreserve BOECs originating from a single colony was considered indicative for its proliferative capacity. Successful cryopreservation of at least one out of three propagated colonies was considered indicative for the proliferative capacity of BOECs derived from a specific subject.

### Immunocytochemistry and fluorescence imaging

Immunocytochemistry and fluorescence imaging of fixed cells was performed as previously described [18] using mouse clonal anti-VWF Rag20 [19] and rabbit polyclonal anti-VE-cadherin (BMS158, eBioscience, San Diego, CA, USA) followed by goat anti-mouse IgG-Alexa 568 and goat anti-rabbit IgG-Alexa 488 (Molecular Probes, Leiden, The Netherlands).

### Intervention study

To study the effect of statin therapy on circulating BOECs we included 10 subjects with the most extended subclinical atherosclerosis. These subjects all had a CAC score above the 80^th^ percentile for age and gender. We compared BOEC colony formation in these subjects at baseline and after at least six months of statin therapy.

### Statistical analyses

Results are expressed as mean ± standard deviation (SD), except if indicated otherwise. Student's t-tests and Chi-square tests were used to calculate differences in baseline characteristics between the groups. Variables that did not show a normal distribution were log transformed before they were analysed.

To determine the efficiency of colony isolation we counted the number of wells that were filled with BOEC colonies at day 7, 14, 21 and 28. The number of colonies per ml of blood used for the isolation was calculated for all samples.

We used a logistic regression model to analyse the relation between the dependent variables “successful isolation” and “cryopreservation”, as a marker of proliferation capacity and the independent variable of the presence of (subclinical) CAD. This model was first adjusted for age and sex and additionally adjusted for smoking. We chose not to correct for other risk factors for CVD, since these factors may contribute to the development of the disease and are therefore intermediate factors leading to CVD. We used an adjusted General Linear Model (GLM) to analyse the relation between the dependent variables “number of colonies” per time point and the independent variable of the presence of (subclinical) CAD. This model was first adjusted for age and sex and additionally adjusted for smoking. Again, additional risk factors were considered intermediate factors leading to CVD and therefore we chose not to correct for those. From the cumulative numbers of colonies per time point we calculated the non-cumulative increase in colony number per time point. For each subject we recorded the maximum non-cumulative increase in colony number and the associated time point in two new variables. For subjects that did not provide any colonies we recorded a maximum non-cumulative increase of zero at time point 35 days. The “time to maximum increase” was obtained by dividing the maximum non-cumulative increase in colony number by the associated time point. A similar GLM as described above was used to analyse these variables. Data in all GLM models was log transformed before the analyses; log(colony number day 28+0.001), log(maximum increase+0.001) and log(time to maximum increase+0.0001), respectively, were used in analyses to account for zero values.

A Wilcoxon signed rank test was used to analyse the number of colonies before and after statin therapy in the intervention study. All analyses were performed using SPSS for Windows 19.0. A p-value <0.05 was considered statistically significant.

## Results

### Subjects

In total 227 individuals met the inclusion criteria, of which 70 were patients, 99 were healthy FDRs and 58 were FDRs with subclinical atherosclerosis. Baseline characteristics are shown in table 1. Patients were significantly more often male compared with FDRs. Furthermore, classical risk factors for CVD were more often present in patients compared with the healthy FDRs. Due to the high percentage of subjects on statin therapy in the patient group, no data on cholesterol levels are provided.

**Table 1:** Baseline characteristics.

	Healthy FDRs	FDRs with subclinical atherosclerosis	Patients
n	99	58	70
Age, years ± SD	41.5±11.2	53.0±9.1 *	55.3±9.8 *
Gender, male (%)	38 (38)	26 (45)	53 (76) * †
Hypercholesterolemia, n (%)	18 (18)	20 (34) *	38 (54) * †
Hypertension, n (%)	13 (13)	23 (40) *	30 (43) *
Diabetes, n (%)	2 (2)	8 (14) *	10 (14) *
Smoking, n (%)	36 (37)	18 (32)	35 (50) †
Statin use, n (%)	11 (11)	19 (33) *	66 (94) * †
BMI, kg/m2 ± SD	25.6±4.2	27.5±3.7 *	27.8±4.1 *
Systolic blood pressure, mmHg ± SD	126±12	135±16 *	132±17 *
Diastolic blood pressure, mmHg ± SD	77±9	83±9 *	80±12 *
Glucose, mmol/L ± SD	5.0±0.6	5.5±1.2 *	5.9±1.8 *
Total cholesterol, mmol/L ± SD	5.3±1.0	5.7±1.0 *	
HDL cholesterol, mmol/L ± SD	1.4±0.4	1.4±0.4	
LDL cholesterol, mmol/L ± SD	3.4±0.9	3.6±0.9	
Triglycerides, mmol/L ± SD	1.1±0.8	1.5±0.8 *	
Successful isolation, n (%)	79 (80)	42 (72)	52 (74)
Colonies at day 28, n [IQR]	0.07 [0–0.15]	0.06 [0–0.11]	0.07 [0–0.19]
Max increase in colony number, n [IQR]	0.04 [0.04–0.11]	0.04 [0–0.07]	0.04 [0–0.11]
Time point max increase, days ± SD	23±8	24±8	22±8
Time to max increase [IQR]	0.003 [0.001–0.005]	0.002 [0–0.003]	0.003 [0–0.006]
Proliferation capacity, n (%)	69 (70)	31 (53) *	43 (61)

Continuous data are expressed as mean ± SD or median [IQR], categorical data as absolute numbers with (percentages).

* p<0.05 compared to healthy FDRs.

† p<0.05 compared to FDRs with subclinical atherosclerosis.

BMI, body mass index; FDRs, first-degree relatives; HDL, high-density lipoprotein; IQR, inter quartile range; LDL, low-density lipoprotein; SD, standard deviation.

### BOECs isolation and proliferation

**Figure 1:**
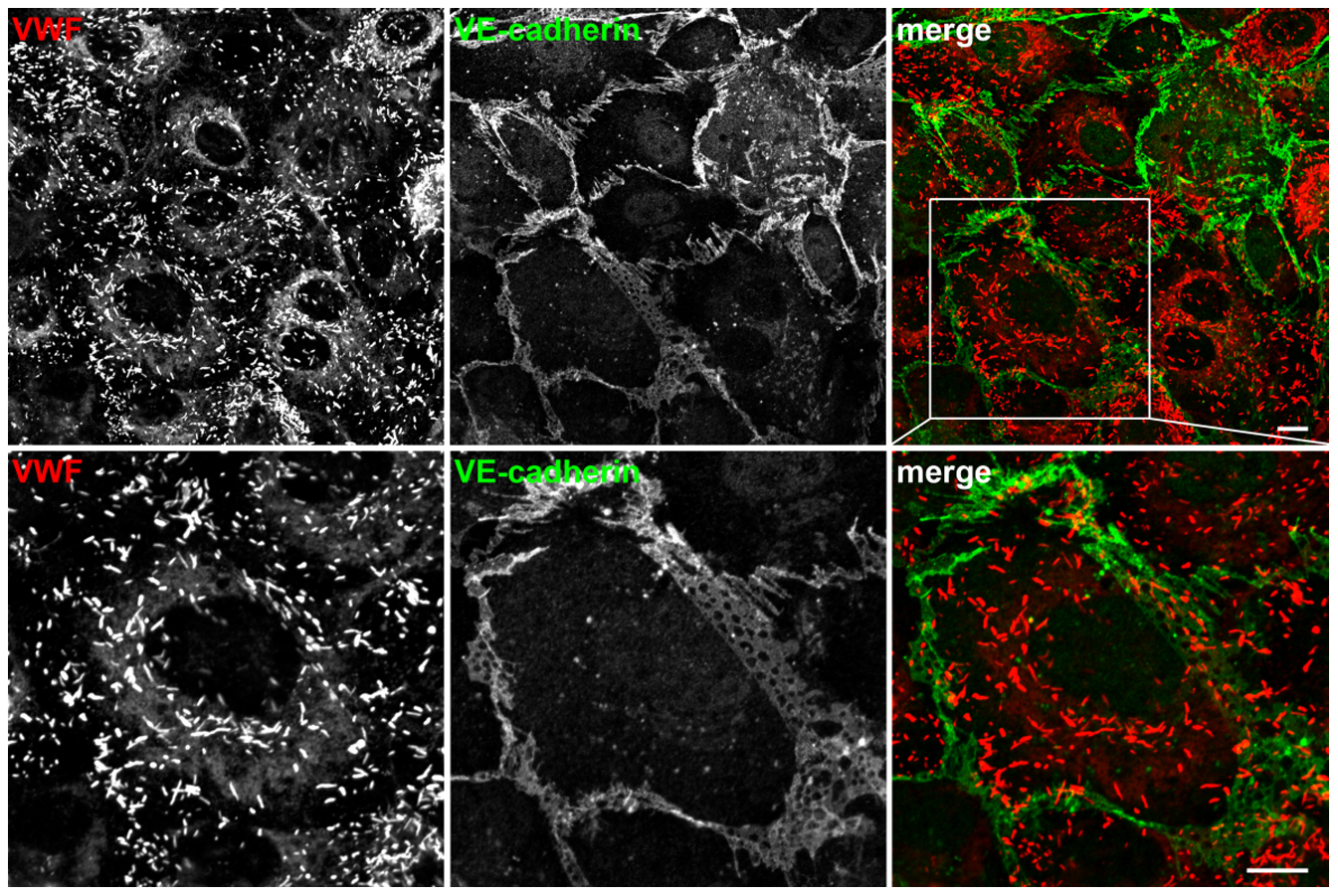
Morphological characterization of blood outgrowth endothelial cells. Paraformaldehyde fixed BOECs were immunostained for VWF (red) and VE-cadherin (green) to delineate Weibel-Palade bodies and endothelial cell boundaries. Magnifications of the boxed region are shown in the bottom row. Scale bars represent 10 µm.

Colonies were cultured from the majority of subjects. Immunocytochemistry and fluorescence imaging revealed that these colonies were BOECs (figure 1).

At baseline, proliferative capacity was significantly impaired in FDRs with subclinical atherosclerosis compared with healthy FDRs (53% vs 70%; p<0.05, table 1). Interestingly, patients showed an intermediate phonotype of 61% proliferative capacity, which is not significantly different compared to either the healthy FDRs or the FDRs with subclinical atherosclerosis (table 1).

An adjusted logistic regression confirmed that the proliferative capacity of BOEC colonies was significantly lower in FDRs with subclinical atherosclerosis compared with healthy FDRs (OR = 0.45; 95%CI 0.21–0.96; p<0.05, table 2). Again, patients showed an intermediate phenotype which was not significantly different compared to either the healthy FDRs of the FDRs with subclinical atherosclerosis. Additionally, we did not observe any differences in the success rate of colony isolation between the three groups (table 2.)

**Table 2:** Relation between colony isolation success rate and proliferation capacity and subclinical or overt CAD.

	Colony isolation		Proliferation capacity	
	OR	95% CI	OR	95% CI
**Healthy FDRs**	1.0		1.0	
**Patients**				
Model 1	0.73	0.35–1.51	0.69	0.36–1.32
Model 2	0.63	0.26–1.53	0.68	0.31–1.49
Model 3	0.54	0.22–1.37	0.63	0.28–1.43
				
**FDRs with subclinical CAD**				
Model 1	0.67	0.31–1.42	0.50	0.26–0.98 *
Model 2	0.58	0.25–1.35	0.47	0.22–0.99 *
Model 3	0.55	0.23–1.29	0.45	0.21–0.96 *
**Patients**			1.0	
**FDRs with subclinical CAD**				
Model 1	0.91	0.41–2.00	0.72	0.36–1.46
Model 2	0.93	0.41–2.10	0.70	0.34–1.45
Model 3	1.01	0.44–2.31	0.72	0.34–1.51

Model 1, crude model; model 2, adjusted for age and sex; model 3, additionally adjusted for smoking. Outcomes are expressed as odds ratios with corresponding 95% confidence intervals * p<0.05. CAD, coronary artery disease; CI, confidence interval; FDRs, first-degree relatives; OR, odds ratio.

To be able to draw any conclusions about the colony forming rate of BOECs it is important to not only analyse the number of colonies, but also the rate at which the colonies are formed. High numbers of BOEC colonies at an early stage represent a highly viable cell culture. Therefore, we calculated the time to maximum increase, by dividing the maximum non-cumulative increase in colony number of colonies by the time point associated with this maximum increase. After adjusting for age, gender and smoking, GLM analysis showed a positive association between the time point of maximum increase and the presence of subclinical atherosclerosis (B = 0.14; 95%CI 0.01–0.27; p<0.05, table 3), meaning that the maximum increase occurs later in FDRs with subclinical atherosclerosis compared with healthy FDRs. Also, we observed a trend towards a negative association between the maximum increase and subclinical atherosclerosis (B = −0.67; 95%CI −1.43–0.008; p = 0.081, table 3), with a lower maximum increase in colony numbers in FDRs with subclinical atherosclerosis compared with healthy FDRs. Together, this resulted in a trend towards a negative association between the time to maximum increase and the presence of subclinical atherosclerosis, meaning that the time to maximum increase seems lower in FDRs with subclinical atherosclerosis compared with healthy FDRs. These analyses again showed an intermediate phenotype in patients (table 3).

**Table 3:** Association between BOEC isolation parameters and the presence of subclinical or overt CAD.

	Colony numbers day 28	Max increase in colony number	Time point of max increase	Time to max increase
**Healthy FDRs**	1.0	1.0	1.0	1.0
**Patients**				
Model 1	−0.30 (−1.00–0.40)	−0.32 (−0.97–0.32)	0.02 (−0.09–0.13)	−0.28 (−0.87–0.32)
Model 2	−0.44 (−1.30–0.42)	−0.45 (−1.25–0.35)	0.03 (−0.11–0.17)	−0.38 (−1.11–0.35)
Model 3	−0.63 (−1.50–0.23)	−0.62 (−1.42–0.19)	0.06 (−0.08–0.20)	−0.55 (−1.28–0.19)
**FDRs with subclinical CAD**				
Model 1	−0.52 (−1.25–0.22)	−0.51 (−1.20–0.17)	0.11 (−0.01–0.23)	−0.53 (−1.16–0.10)
Model 2	−0.65 (−1.47–0.17)	−0.63 (−1.39–0.13)	0.13 (0.00–0.26)	−0.64 (−1.33–0.06)
Model 3	−0.70 (−1.51–0.11)	−0.67 (−1.43–0.08)	0.14 (0.01–0.27)*	−0.68 (−1.37–0.02)
**Patients**	1.0	1.0	1.0	1.0
**FDRs with subclinical CAD**				
Model 1	−0.22 (−101–0.58)	−0.19 (−0.93–0.55)	0.09 (−0.04–0.22)	−0.26 (−0.93–0.42)
Model 2	−0.22 (−1.04–0.61)	−0.18 (−0.94–0.59)	0.10 (−0.03–0.23)	−0.26 (−0.96–0.45)
Model 3	−0.07 (−0.89–0.76)	−0.05 (−0.82–0.72)	0.08 (−0.05–0.21)	−0.13 (−0.84–0.57)

Model 1, crude model; model 2, adjusted for age and sex; model 3, additionally adjusted for smoking. Outcomes are expressed as B with corresponding 95% confidence intervals * p<0.05.

FDRs, first-degree relatives.

### Intervention study

**Figure 2:**
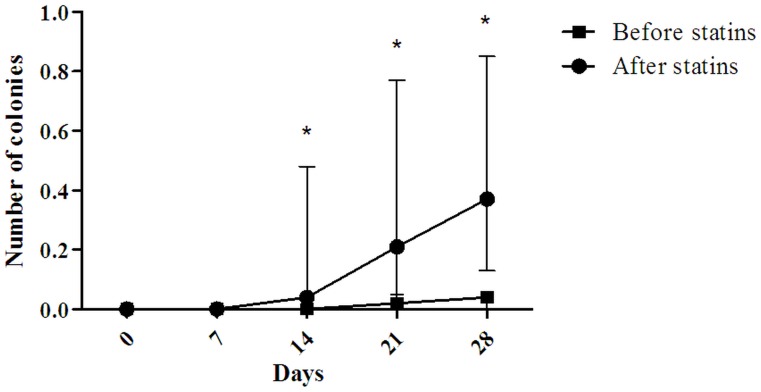
Colony formation curves of FDRs with subclinical atherosclerosis before and after statin treatment. This graph shows the median number of colonies at specific time points before and after statin therapy. Error bars indicate de inter quartile ranges. * P<0.05.

To study the effect of statin therapy on circulating BOEC precursors we analysed BOEC colony formation before and after statin therapy in ten subjects with severe subclinical atherosclerosis, as assessed by coronary CT-scanning. From day 14 on, we saw a significant increase in the number of BOEC colonies after statin therapy (figure 2). Not only the number of colonies at day 28 (0.04 [0–0.06] vs. 0.37 [0.13–0.85]; p<0.05), but also the maximum increase in colony number (0.04 [0–0.05] vs. 0.26 [0.07–0.59]; p<0.05) and the time to maximum increase (0.002 [0–0.003] vs. 0.013 [0.005–0.034]; p<0.05) were significantly increased after statin therapy. Also, the proliferative capacity was increased after statin therapy (30% vs 80%, table 4), but due to small sample size, this did not reach statistical significance.

**Table 4:** BOEC isolation parameters and proliferation capacity in subclinical CAD before and after statin therapy.

	Before treatment	After treatment
Successful isolation, n (%)	6 (60)	9 (90)
Colonies at day 28, n [IQR]	0.04 [0–0.06]	0.37 [0.13–0.85] *
Max increase in colony number, n [IQR]	0.04 [0–0.05]	0.26 [0.07–0.59] *
Time point max increase, days ± SD	26.60±7.95	21.00±8.08
Time to max increase [IQR]	0.002 [0–0.003]	0.013 [0.005–0.034] *
Proliferation capacity, n (%)	3 (30)	8 (80)

Continuous data are expressed as mean ± SD or median [IQR], categorical data as absolute numbers with (percentages).

* p<0.05 compared with before treatment.

IQR, inter quartile range; SD, standard deviation.

## Discussion

In this study we show that BOECs isolated from apparently healthy FDRs with subclinical atherosclerosis are less viable, resulting in a decreased proliferative capacity compared with both healthy FDRs and patients with premature CAD. Interestingly, parameters of BOEC isolation and proliferation of premature CAD patients did not differ from either FDRs with subclinical atherosclerosis or from healthy FDRs, indicating an intermediate phenotype. This is interesting, since the both patients and FDRs with subclinical atherosclerosis have atherosclerotic disease, and one would have expected a similar proliferative capacity. On the other hand, statin therapy could have restored the proliferative capacity, as observed in our second part of the study. This however, was not the case. The fact that the proliferative capacity was not completely restored, could be due to the fact that not all patients adhered to their treatment, which resulted in the intermediate phenotype. Otherwise, subclinical atherosclerosis and full blown CAD are not completely similar, thus the atherosclerotic burden in combination with the restorative capacity of statin therapy might have resulted in an intermediate phenotype. Finally, differences might also have been non-significant due to limited power. However, in our opinion the comparison between both groups of FDRs is the least confounded, since both groups were initially perceived as healthy individuals, and therefore this would be the best comparison.

To further explore the restorative capacity of statin therapy, we additionally analyzed this before and after statin therapy in a subgroup of FDRs with subclinical atherosclerosis.

Indeed, we observed a strong increase in BOEC colony formation after statin treatment in FDRs with subclinical atherosclerosis.

In previous studies similar results have been reported for eEPCs, which were shown to be a useful biomarker for CVD [3], with lower levels of eEPCs in CVD patients compared with healthy controls [4], [6], [20]. Additionally, eEPC levels could independently predict disease progression [14], and already in subjects with subclinical atherosclerosis, as identified by carotid inter media thickness and coronary CT scanning, reduced eEPC levels were detected [21], [22]. However, there is a major difference between eEPCs and BOEC cell colonies. eEPCs belong to the haematopoietic lineage, partially retaining their myeloid progenitor activity and explicitly do not resemble endothelial cells. Although these cells can facilitate vasculogenesis *in vivo*, they do not have the ability to form secondary endothelial colonies [23]. On the contrary, BOECs are highly proliferative cells that express solely endothelial cell markers [24] and therefore, these cells have been described as “true” EPCs [25]. Only few studies have assessed characteristics of BOECs in relation to CVD and revascularization. In a recent study it was shown that after an acute coronary event BOECs could be cultured only from a minority of the patients [26]. The ability to culture BOECS from these patients was associated with better outcomes in terms of reduced microvascular obstruction and reduction of infarct size [27]. In contrast, Massa et al. was not able to show a difference in BOEC cultures between CAD patients and healthy controls [28]. However, the presence of subclinical atherosclerosis was not assessed in the controls in this study, which might have confounded the results. Additionally, due to the small sample size, small but clinically relevant differences between patients and controls might have been missed. Here we show that in subjects with subclinical atherosclerosis there is a trend towards lower levels of circulating BOEC precursors and that the proliferative capacity is significantly impaired compared to their healthy relatives. This may possibly affect the potential of vascular regeneration in these subjects, thereby increasing the risk of CVD. Treatment with statins potentially improved BOEC colony formation and proliferation in our group of premature CAD patients, resulting in an intermediate phenotype. Different animal models have shown that statins, apart from there lipid lowering effect, increased mobilization of EPCs [29] and improved neovascularization in experimentally induced myocardial infarction [30]. Now we show that also BOEC colony formation and proliferation significantly increases in subjects with subclinical atherosclerosis after statin therapy. Whether this subsequently results in an improved endothelial repair and a decreased incidence of coronary events in these subjects remains to be investigated.

### Study strengths and limitations

There are some potential limitations of the design of this study that have to be addressed. First, subjects below the age of 30, in whom no CT-scanning was performed, were considered healthy controls. However, these individuals could already have developed subclinical atherosclerosis which could have negatively influenced the numbers of BOEC colonies in the group of healthy FDRs, resulting in an underestimation of the differences between the study groups. Second, the number of BOEC colonies is generally low and the differences between the study groups are small. As a consequence, our sample size was only marginally sufficient to show significant differences in parameters of BOEC isolation. However, we did show consistent trends towards a decreased viability of BOEC colonies. Therefore, we believe that this is not an incidental finding, but a real phenomenon.

Strength of this study is the division of the FDRs in FDRs with subclinical atherosclerosis and truly healthy FDRs based on coronary CT scanning. By doing so, we made sure that results in our control population are not influenced by the presence of asymptomatic disease. Also, this enabled us to show that, already during the development of atherosclerotic disease endothelial proliferation is impaired. Based on this we hypothesize that circulating BOEC precursors can be used as a biomarker for subclinical atherosclerosis in apparently healthy individuals. Meneveau and co-workers have shown that the level of circulating CD34+/VEGF-R2+ cells correlates with the number of BOEC colonies in patients with acute myocardial infarction. Their findings also suggest that the presence of BOECs correlated with preserved microvascular integrity following an myocardial infarction [27]. Together with the results reported in our study this suggests that circulating BOEC precursors contribute to the regenerative capacity of the vasculature, thereby reducing the propensity of developing CVD.
